# Editorial: Assessing the power of HIV self-testing in unreachable populations in sub-Saharan Africa

**DOI:** 10.3389/fpubh.2022.1078729

**Published:** 2022-11-15

**Authors:** Joseph K. B. Matovu, Augustine T. Choko, Jeffrey E. Korte, Donaldson F. Conserve

**Affiliations:** ^1^Department of Disease Control and Environmental Health, Makerere University School of Public Health, Kampala, Uganda; ^2^Department of Community and Public Health, Busitema University Faculty of Health Sciences, Mbale, Uganda; ^3^Public Health Group, Malawi Liverpool Wellcome Trust Clinical Research Programme, Blantyre, Malawi; ^4^Department of Public Health Sciences, Medical University of South Carolina, Charleston, SC, United States; ^5^Department of Prevention and Community Health, Milken Institute School of Public Health, The George Washington University, Washington, DC, United States

**Keywords:** HIV self-testing, unreachable populations, sub-Saharan Africa, key populations, men

## Introduction

HIV self-testing has been touted as the game-changer in HIV testing uptake with high HIV testing rates reported in studies conducted in low and middle-income countries, a majority of which were conducted in sub-Saharan Africa (1). In this highly successful Research Topic, we assessed the power of HIV self-testing in reaching unreachable populations in sub-Saharan Africa. Overall, 17 papers have been published in response to this Research Topic from eight countries (Mali, Senegal, Cote d'Ivoire, Ghana, South Africa, Uganda, Zimbabwe, and the Democratic Republic of Congo); 14 papers in volume 1 and three papers in volume 2. The papers published as part of this Research Topic demonstrate that HIV self-testing has the power to reach a diversity of unreachable populations including men who have sex with men (MSM), female sex workers (FSW), people who use drugs (PWUDs), truck drivers, men in the general population, and other unreachable populations. This Research Topic has received a high level of visibility with over 41,000 views by October 23, 2022.

## Key and priority populations

Evidence shows that HIV testing uptake among key populations (KPs) remains sub-optimal despite the fact that these populations contribute up to 51% of new HIV infections in sub-Saharan Africa (2). Four studies reported on different approaches used to reach KPs with HIV self-testing services. Abubakari et al. used community-based interventions, enhanced with mobile platforms and digital technology, as opportunities to increase HIV self-testing and linkage to HIV care among MSM in Ghana. Abubakari et al. worked with community-based organization partners to implement three interventions that successfully engaged and retained MSM which provided an opportunity for linkage to HIV self-testing and medical care. d'Elbee estimated the cost of integrating HIV self-testing into 23 civil society organization (CSO)-led models for key populations in Senegal, Cote d'Ivoire, and Mali and found that providing HIV self-test kits to KPs through CSOs was not only cost-effective but had varying levels of cost-effectiveness. The team found that the cost of reaching female sex workers with HIV self-testing services was much lower than that for reaching MSM and PWUDs (FSW: $13–17; MSM: $15–28; PWUDs: $16–144). Okoboi et al. found that the cost per new HIV-positive MSM identified ($325 vs. 914) and the cost per HIV transmission averted ($6,253 vs. 17,567) through HIV self-testing was much lower than the cost per new HIV-positive MSM identified and new HIV transmissions averted through conventional HIV testing services. The team concluded that HIVST was not only cost-effective but also identified more undiagnosed HIV infections than standard-of-care HIV testing. Kra et al. described the adaptations that the HIV self-testing teams used to navigate the challenges posed by the COVID-19 pandemic including the use of social networks by MSM peer educators to maintain contact with their peers, promote HIV prevention and testing, and organize face-to-face or small group meetings, as needed. These adaptations were essential for the continued provision of HIV self-testing services during the COVID-19 lockdown in Mali, Senegal, and Cote d'Ivoire.

## Truck drivers

Mantell et al. and Kelvin et al. found that truck drivers preferred blood-based HIV testing over oral-based HIV self-testing. In the study by Kelvin et al. 305 truck drivers were randomized to receive oral HIV self-test kits or stand-of-care HIV testing and followed up for 6 months. At the end of the follow-up period, HIV testing uptake was similar [56.3% in the intervention arm and in the standard-of-care arm (55.6%)], with those who did not test for HIV in both arms citing reasons related to lack of time to test for HIV, low HIV risk behavior, fear of knowing their HIV status and recent HIV testing. When asked to choose between blood-based and oral HIV self-testing, 69.4% preferred blood-based HIV testing. Similar results were reported by Sithole et al. who found that of the men who were given the option to choose between oral or blood-based HIV self-test kits, 62% (1,624) preferred to use the blood-based kits while 38% (1,010) selected to use the oral fluid kits; suggesting a growing interest in blood-based HIV self-test kits. This interest is usually driven by beliefs, particularly among men, that since HIV is found in blood, then, blood-based HIV self-testing strategies could yield the most realistic results (3).

## Men in the general population

Men in the general population have been dubbed as the missing link in HIV prevention programming. Three studies assessed approaches for reaching men with HIV self-testing services. Sithole et al. used community-based recruitment procedures, including distributing HIV self-test kits at venues where men were likely to congregate, e.g., taxi pranks, to reach men with HIV self-testing services in KwaZulu-Natal, South Africa. The team found that reaching men in places where they congregate was not only feasible but also highly effective in reaching men, including those who had not previously tested for HIV. However, Tonen-Wolyec et al. found that linkage to HIV care was much lower in men than women, suggesting that despite the increasing HIV testing rates and identification of new HIV-positives as a result of HIV self-testing, additional innovative approaches are still needed to improve linkage to HIV care among men who self-test HIV-positive. Muwanguzi et al. found that the use of phone reminders; consistent, open and regular communication with the research team; providing HIV-positive men with an enabling, non-stigmatizing health environment; the ease with which HIV-positive men with referral forms were attended to by health workers, and trust that health workers would keep their HIV-positive status confidential, facilitated HIV-positive men to link to HIV care. Future studies should assess the extent to which a combination of these interventions can help to enhance linkage to HIV care among men who are reluctant to link to HIV care or if they do so, they link to HIV care late, usually with advanced HIV disease.

## Other populations

Amstutz et al. assessed the cost of reaching absent or refusing individuals through provision of HIV self-testing services in the intervention arm, as part of a home-based, randomized controlled HIV testing intervention in rural Lesotho. The team concluded that adding HIV self-testing to conventional HIV testing services not only increased HIV testing coverage by 21% but also reduced the cost per person tested. Sithole et al. used 63 men and women living with HIV participating in an HIV treatment trial in KwaZulu-Natal, South Africa, as HIV self-test kits distributors to reach their social and sexual networks. HIV self-test kits distributors took 218 kits; of these, 143 (65.6%) were reported as used by their recipients. Forty-two per cent of the testers were first-time testers. However, linkage to HIV care remained low with only 9% of the 11 HIV-positive individuals identified were linked to HIV care. McGowan et al. explored PrEP naïve and PrEP-experienced adolescent girls and young women's (AGYW) willingness to engage in a peer-delivered HIV self-testing and referral model for PrEP initiation in Kiambu County, Kenya. Study findings show that PrEP-experienced AGYW were willing to initiate discussions about HIV self-testing and PrEP use among their peers, to deliver HIV self-test kits to them, and to refer them to appropriate HIV prevention, care and treatment services, based on their HIV status. PrEP-naïve AGYW were also willing to receive and use HIV self-test kits delivered to them by their peers and to link to appropriate HIV prevention, care and treatment services based on their HIV test results. Muchedzi et al. distributed 11,983 kits between 2018 and 2020 in Zimbabwe; of these, 99.5% (11,924/11,983) were used and results were returned to the health care workers. Of the returned HIVST results, 22.3% (2,658/11,924) were reactive and, of these, 2,610 (98.2%) results were confirmed HIV positive by a trained health care worker using the national testing algorithm. The highest positivity rate was reported among users aged 35–49 years (25.5%, *n* = 667). The prevalence of HIV in the study population was nearly twice as high as the prevalence reported among adults 15 years or older in the 2020 Zimbabwe Population-based HIV Impact Assessment survey (22.3 vs. 12.9%) (4).

## Moving the HIV self-testing agenda forward

Evidence from two papers, published as part of this Research Topic, shows that policymakers and other key stakeholders consider HIV self-testing to be an opportunity to reduce stigma; preserve anonymity and confidentiality; reach key populations that do not access HIV testing *via* conventional HIV testing strategies; remove spatial barriers; save time for users and providers; and empower users with autonomy and responsibility (Nagai et al.; Ky-Zerbo et al.). However, as Ky-Zerbo et al. reported, stakeholders doubted potential HIVST users' autonomy regarding their ability to use HIVST kits correctly; to ensure quality secondary distribution; to accept a reactive test result; and to use confirmation testing and care services. Similar sentiments have been reported in other settings where HIVST interventions are being introduced for the first time (5–7) and suggest a need for pre-project implementation stakeholder meetings to identify and address such fears as part of HIVST project initiation activities. Due to the low partner HIV status disclosure among PLHIV (Boye et al.), future interventions will need to enhance HIV disclosure as part of HIV self-testing promotional strategies, especially in populations with traditionally low HIV disclosure rates. As Hamilton et al. have argued, probably there is no one intervention strategy that will work universally to increase HIV testing uptake and linkage to appropriate HIV prevention or care and treatment services. Multiple interventions will be needed to reach men and other unreachable populations with HIV self-testing services including peer-to-peer distribution, use of community health counselors, use of trained lay distributors selected by the community, and integration of HIV self-testing services into other HIV services. Also, multiple interventions will be needed to enhance linkage to HIV care including home-based ART initiation, use of phone reminders, and community-based ART initiation.

## Author contributions

JKBM wrote the initial draft. All authors contributed to the article and approved the submitted version.

## Conflict of interest

The authors declare that the research was conducted in the absence of any commercial or financial relationships that could be construed as a potential conflict of interest.

## Publisher's note

All claims expressed in this article are solely those of the authors and do not necessarily represent those of their affiliated organizations, or those of the publisher, the editors and the reviewers. Any product that may be evaluated in this article, or claim that may be made by its manufacturer, is not guaranteed or endorsed by the publisher.
